# Metabolic Signatures of Type 2 Diabetes Mellitus and Hypertension in COVID-19 Patients With Different Disease Severity

**DOI:** 10.3389/fmed.2021.788687

**Published:** 2022-01-10

**Authors:** Mohamed A. Elrayess, Farhan S. Cyprian, Abdallah M. Abdallah, Mohamed M. Emara, Ilhame Diboun, Najeha Anwardeen, Sven Schuchardt, Hadi M. Yassine

**Affiliations:** ^1^Biomedical Research Center (BRC), Qatar University, Doha, Qatar; ^2^Department of Basic Medical Sciences, College of Medicine, QU Health, Qatar University, Doha, Qatar; ^3^Biomedical and Pharmaceutical Research Unit, QU Health, Qatar University, Doha, Qatar; ^4^College of Health and Life Sciences, Hamad Bin Khalifa University (HBKU), Doha, Qatar; ^5^Department of Bio- and Environmental Analytics, Fraunhofer Institute for Toxicology and Experimental Medicine (ITEM), Hannover, Germany; ^6^College of Health Sciences, Qatar University, Doha, Qatar

**Keywords:** COVID-19, diabetes mellitus, hypertension, triacylglycerols, palmitic acid, docosapentaenoic acid, docosahexaenoic acid, oleic acid

## Abstract

**Introduction:** Increased COVID-19 disease severity is higher among patients with type 2 diabetes mellitus and hypertension. However, the metabolic pathways underlying this association are not fully characterized. This study aims to identify the metabolic signature associated with increased COVID-19 severity in patients with diabetes mellitus and hypertension.

**Methods:** One hundred and fifteen COVID-19 patients were divided based on disease severity, diabetes status, and hypertension status. Targeted metabolomics of serum samples from all patients was performed using tandem mass spectrometry followed by multivariate and univariate models.

**Results:** Reduced levels of various triacylglycerols were observed with increased disease severity in the diabetic patients, including those containing palmitic (C16:0), docosapentaenoic (C22:5, DPA), and docosahexaenoic (C22:6, DHA) acids (FDR < 0.01). Functional enrichment analysis revealed triacylglycerols as the pathway exhibiting the most significant changes in severe COVID-19 in diabetic patients (FDR = 7.1 × 10^−27^). Similarly, reduced levels of various triacylglycerols were also observed in hypertensive patients corresponding with increased disease severity, including those containing palmitic, oleic (C18:1), and docosahexaenoic acids. Functional enrichment analysis revealed long-chain polyunsaturated fatty acids (n-3 and n-6) as the pathway exhibiting the most significant changes with increased disease severity in hypertensive patients (FDR = 0.07).

**Conclusions:** Reduced levels of triacylglycerols containing specific long-chain unsaturated, monounsaturated, and polyunsaturated fatty acids are associated with increased COVID-19 severity in diabetic and hypertensive patients, offering potential novel diagnostic and therapeutic targets.

## Introduction

The coronavirus disease 2019 (COVID-19) pandemic remains a major challenge to global healthcare. COVID-19 is categorized into different levels of severity depending on the clinical manifestations of the disease (1). Most COVID-19 patients (~80%) have asymptomatic or mild infections that require little to no medical intervention (2). However, up to 20% of COVID-19 patients develop interstitial pneumonia and respiratory failure that demands intensive care, especially among older individuals and those with chronic illnesses (3, 4). Previous reports have shown that there is a high risk of lung infection in patients with type 2 diabetes mellitus (T2DM) (5). Respiratory disease in T2DM patients usually progresses to a severe form in a short period of time, making T2DM a risk factor for mortality in COVID-19 patients. Furthermore, accumulating evidence suggests that COVID-19 is more prevalent among individuals suffering from T2DM and hypertension (6–8), with studies showing that treatment of these patients with angiotensin-converting enzyme 2 (ACE2)-increasing drugs could lead to a severe outcome (9).

The progression of COVID-19 from mild to more severe forms is associated with systemic changes in metabolism, and such COVID-induced metabolic alterations have been the subject of wide investigation. Various metabolic biomarkers have been identified with potential diagnostic, prognostic, and therapeutic value for COVID-19 patients (10–12). In terms of COVID-19 severity, metabolites such as kynurenine, sphingolipids (10, 11), and anthracitic acid (12) were identified as potential biomarkers.

Despite multiple studies addressing COVID-19 metabolomics (10, 13, 14), no study has addressed the metabolic profiling of COVID-19 patients with T2DM and hypertension. The objective of this study was to investigate the metabolic signatures associated with diabetic and hypertensive COVID-19 patients as well as different levels of disease severity. Identifying potential diagnostic biomarkers could further the understanding of the underlying molecular mechanisms responsible for increased risk of mortality in COVID-19 patients.

## Methods

### Study Design

**Figure 1:**
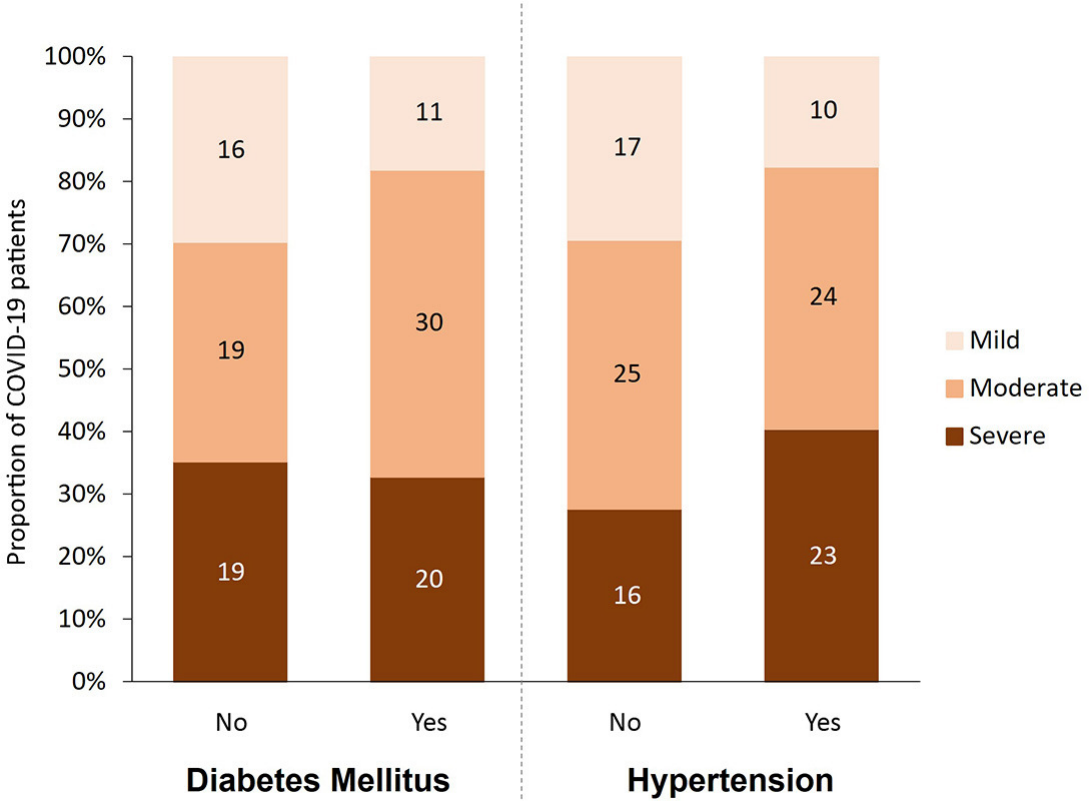
Participants categorized by disease severity (mild, moderate and severe), diabetes status (yes, no), and hypertension status (yes, no).

This cohort study included 115 patients containing type 2 diabetic (*n* = 61, 53%) and non-diabetic (*n* = 54, 47%) patients diagnosed with COVID-19 at Hamad Medical Corporation (HMC) between July and December 2020. The patients were predominantly males based on COVID-19 exposure with an age ranging between 48 and 69 years from diverse nationalities. Protocols were approved by the Institutional Review Boards (IRBs) of HMC (MRC-01-20-145) and Qatar University (QU-IRB 1289-EA/20). The cohort of COVID-19 patients in this study were categorized using WHO classification of clinical presentation as severe (SpO2 <94% on room air), moderate (radiographic pulmonary involvement, fever, respiratory symptoms) and mild/asymptomatic (no clinical finding only RT-PCR positive cases). Accordingly, participants were divided into three groups based on disease severity [mild/asymptomatic (*n* = 27), moderate (*n* = 49), and severe (*n* = 39)], and further dichotomized into non-diabetic and diabetic patients and into normotensive and hypertensive patients. Figure 1 shows the breakdown of participants in each of the studied groups. Blood samples were collected at the time of diagnosis, prior to isolation, or hospitalization. COVID-19 patients with moderate to severe disease were hospitalized for inpatient management, out of which 39 cases were admitted to ICU and blood samples were taken at the time of admission. In the severe group, 18 patients died with respiratory failure listed as the primary cause of death. Clinical and laboratory data including Body Mass Index (BMI), viral load, routine and specific blood tests were retrieved from hospital's electronic healthcare system following patients consent.

### Metabolomics

Targeted metabolomics of serum samples collected from all participants between 24 and 48 h after diagnosis was conducted using Biocrates MxP^®^ Quant 500 Kit (Biocrates, Innsbruck, Austria) measured by tandem mass spectrometry at Fraunhofer Institute for Toxicology and Experimental Medicine. Six hundred and thirty metabolites were assessed as part of the MetIDQ™ MetaboINDICATOR™ module designed specifically for MxP^®^ Quant 500 kit data. Two hundred and two (202) metabolite indicators consisting of combinations of metabolite measurements that capture meaningful biological functions were also derived. Examples of such indicators include enzymatic activity based on substrate/product metabolite ratio and the sum of functionally/structurally similar metabolites. Flow injection Analysis Tandem Mass Spectrometry (FIA-MS/MS) was used to quantify lipids, and liquid chromatography-tandem mass spectrometry (LC-MS/MS) was used to quantify small molecules using 5500 QTRAP^®^ instrument triple quadrupole mass spectrometer (AB Sciex, Darmstadt, Germany) as described previously (15). For the listed triacylglycerols, the “_” indicates that the positions (sn-1/sn-2/sn-3) of the fatty acid residues are unknown. Potential isomers are described by the manufacturer: https://biocrates.com/wp-content/uploads/2020/02/Biocrates_Q500_isomers_isobars.pdf.

### Statistical Analysis

Multivariate analysis was carried out by performing orthogonal partial least squares discriminant analysis (OPLS-DA) on log-transformed metabolomics data using the software SIMCA (v.16). A linear model was constructed using R version 4.0.3, and each metabolite was depicted as a y-variable against diabetic status and COVID-19 se-verity (mixed model). The interactions between metabolites, diabetes status, and COVID-19 severity were corrected for the confounders of age, gender, BMI and hypertension. A similar linear model was performed for assessing the metabolites associated with hypertensive individuals. COVID-19 severity was included as an ordinal variable with mild (1), moderate (2) and severe (3) categories in both the linear models. For both the diabetic and hypertensive phenotypes, the model was duplicated with COVID-19 severity included as unordered categories. At each fixed level of COVID-19 severity, the marginal means between diabetics/non-diabetics and normotensives/hypertensive were assessed using the R Emmeans package. The nominal *p*-values were corrected for multiple testing using the false discovery rate (FDR) method. The metabolite classes were defined by Biocrates and those with more than three numbers were filtered for the enrichment analysis. Functional enrichment analysis was performed on metabolite lists (ordered by *p*-value) from the linear models using one-way Wilcoxon rank-sum test and then corrected for multiple testing by the FDR method.

## Results

### Clinical Characteristics of Participants

As shown in Table 1, 115 predominantly male COVID-19 patients were classified into mild, moderate, and severe groups according to the severity of their symptoms. As expected, death incidents were significantly higher in the severe group (46.2%) compared to their moderate (2%) and mild (0%) counterparts (*p* < 0.001). Diabetes, hypertension, and their combination, were more prevalent among severe and moderate patients compared to their mild counterparts (*p* < 0.05), although comparing the differences between disease (diabetes and hypertension) and their control groups by severity did not show statistical significant. Out of the 61 COVID-19 patients with diabetes, 50 (82%) showed moderate or severe symptoms, whereas only 38 out of 54 (70%) non-diabetic patients exhibited moderate or severe symptoms (Figure 1). Similarly, 47 out of 57 (82.5%) hypertensive patients suffered from moderate and severe COVID-19 infection compared to only 41 out of 58 (70.6%) normotensive patients (Figure 1). Additionally, 38 (33%) had both diabetes and hypertension, whereas 23 (20%) had diabetes only and 19 (16.5%) had hypertension only, whereas 35 (30%) patients had neither (Table 1). As expected, several factors were significantly altered (increased or decreased) with disease severity, including Age, CRP, high-sensitivity Troponin-T, WBC count, RBC count, Hgb, % Hct, MCV, ANC, % Neutrophil, lymphocyte count, % lymphocyte, % monocyte, % RDW-CV, PT, D-Dimer, Urea, albumin, ALP, Calcium and adjusted calcium, LDH, Ferritin, Eosinophil, Chloride, Potassium, % Basophil, MPV, IL-6, Procalcitonin, Sodium, AST, INR, APT, Triacylglycerole, MCH and Bilirubin (Table 1).

**Table 1:** Demographic characteristics and clinical laboratory values of COVID-19 patients by disease severity.

	**Mild, *n* = 27**	**Moderate, *n* = 49**	**Severe, *n* = 39**	***p*-value**
Living Status				**<0.001**
Alive	27 (28%)	48 (50%)	21 (22%)	
Dead	0 (0%)	1 (5.3%)	18 (95%)	
Age [years]	53 (50–57)	52 (48–60)	61 (57–69)	**<0.001**
BMI (Kg/m^2^)	25.84 (23.31–27.23)	28.36 (26.07–33.3)	27.78 (24.43–32.22)	0.247
T-2-Diabetes Mellitus				0.2
No	16 (30%)	19 (35%)	19 (35%)	
Yes	11 (18%)	30 (49%)	20 (33%)	
Hypertension				0.2
No	17 (29%)	25 (43%)	16 (28%)	
Yes	10 (18%)	24 (42%)	23 (40%)	
T-2-Diabetes and Hypertension				0.168
No	11 (37.9%)	14 (28.5%)	10 (25.6%)	
Yes	5 (18.5%)	19 (38.7%)	14 (35.8%)	
COVID-19 Average CT	21.9 (17.5–27.4)	26.4 (21.6–31.8)	26.4 (22.0–28.9)	0.071
Body Mass Index (BMI) [kg/m^2^]	25.8 (23.3–27.2)	28.4 (26.2–33.5)	27.3 (25.4–29.9)	0.13
Hemoglobin A1C [%]	7.20 (5.75–10.10)	7.20 (6.08–10.00)	6.80 (5.75–8.35)	0.7
Glucose [mmol/L]	6.0 (5.4–8.0)	6.7 (5.3–8.9)	8.1 (6.6–10.2)	0.092
Cholesterol [mmol/L]	4.49 (4.24–4.74)	3.90 (3.53–5.24)	3.53 (3.00–4.85)	0.4
Triacylglycerole [mmol/L]	3.20 (2.00–6.34)	1.32 (1.10–1.80)	1.90 (1.33–2.82)	**0.03**
Low-density lipoprotein (LDL) [mmol/L]	2.77 (2.77–2.77)	2.13 (1.95–2.69)	1.81 (1.57–2.16)	0.5
C-reactive protein (CRP) [mg/L]	3 (2–11)	14 (4–58)	64 (24–114)	**<0.001**
Procalcitonin [ng/mL]	0.14 (0.08–0.20)	0.16 (0.08–0.37)	0.47 (0.17–1.00)	**0.007**
High-sensitivity Troponin-T [ng/mL]	6 (6–88)	10 (7–10)	36 (12–138)	**<0.001**
White blood cell count (WBC) [× 10^3^/μL]	6.3 (5.3–7.1)	6.2 (4.4–9.2)	11.6 (7.8–14.8)	**<0.001**
Red blood cell count (RBC) [× 10^6^/μL]	5.10 (4.73–5.38)	4.90 (4.60–5.30)	3.60 (2.90–4.20)	**<0.001**
Hemoglobin (Hgb) [g/dL]	14.35 (13.50–15.10)	13.60 (12.40–14.70)	9.80 (8.55–12.00)	**<0.001**
Hematocrit (Hct) [%]	43 (40–44)	41 (38–44)	31 (26–36)	**<0.001**
Mean corpuscular volume (MCV) [fL]	86 (82–89)	83 (76–87)	90 (86–93)	**<0.001**
Mean cell hemoglobin (MCH) [pg]	28.55 (27.68–29.75)	27.90 (25.50–29.70)	29.50 (27.90–30.60)	**0.03**
Mean corpuscular hemoglobin concentration (MCHC) [g/dL]	33.45 (33.12–34.08)	33.30 (32.20–34.10)	32.60 (31.85–33.55)	0.079
Absolute neutrophil count (ANC) [× 10^3^/μL]	3.7 (2.5–4.7)	3.5 (2.4–5.8)	10.6 (6.3–12.7)	**<0.001**
Neutrophil [%]	58 (51–66)	60 (52–73)	88 (76–91)	**<0.001**
Lymphocyte count [× 10^3^/μL]	1.70 (1.52–2.32)	1.60 (1.20–2.00)	0.70 (0.45–1.30)	**<0.001**
Lymphocyte [%]	30 (25–36)	29 (20–38)	7 (4–12)	**<0.001**
Monocyte count [× 10^3^/μL]	0.60 (0.50–0.69)	0.50 (0.40–0.70)	0.60 (0.30–0.85)	0.9
Monocyte [%]	8.6 (7.1–10.9)	8.2 (6.4–9.8)	5.3 (3.1–7.0)	**<0.001**
Eosinophil count [× 10^3^/μL]	0.10 (0.00–0.20)	0.00 (0.00–0.10)	0.00 (0.00–0.10)	0.2
Eosinophil [%]	1.20 (0.45–2.92)	0.80 (0.10–1.60)	0.20 (0.00–0.90)	**0.004**
Basophil count [× 10^3^/μL]	0.030 (0.020–0.040)	0.020 (0.010–0.050)	0.030 (0.010–0.050)	0.7
Basophil [%]	0.50 (0.30–0.67)	0.30 (0.20–0.70)	0.20 (0.15–0.40)	**0.006**
Platelet [× 10^9^/L]	236 (222–282)	256 (201–324)	256 (193–354)	>0.9
Mean platelet volume (MPV) [fl]	10.10 (9.72–10.65)	10.35 (9.78–11.30)	11.00 (10.17–11.90)	**0.006**
Platelet distribution width (PDW) [fl]	15.10 (13.65–15.55)	13.00 (11.20–15.88)	12.85 (11.67–15.30)	0.8
Red blood cell distribution width (RDW-CV) [%]	12.35 (11.93–12.97)	13.30 (12.60–14.60)	14.90 (13.80–17.65)	**<0.001**
Prothrombin time (PT) [second]	11.75 (11.33–12.17)	11.20 (11.05–11.70)	13.50 (12.33–14.95)	**<0.001**
International Normalized Ratio (INR)	1.05 (1.02–1.08)	1.00 (1.00–1.00)	1.10 (1.00–1.30)	**0.023**
D-Dimer [mg/L FEU]	0.88 (0.54–1.21)	0.50 (0.36–1.07)	2.53 (1.28–5.28)	**<0.001**
Fibrinogen [g/L]	6.80 (6.80–6.80)	4.60 (4.60–4.60)	3.90 (3.10–4.90)	0.2
Partial thromboplastin time (APTT) [second]	26 (26–26)	29 (27–32)	33 (28–39)	**0.026**
Urea [mmol/L]	4 (3–5)	5 (4–6)	13 (7–24)	**<0.001**
Creatinine [μmol/L]	82 (69–90)	78 (62–92)	83 (64–134)	0.2
Bilirubin [mg/dL]	10 (5–12)	7 (5–12)	10 (8–14)	**0.034**
Total Protein [g/L]	76 (70–78)	71 (67–73)	68 (58–75)	0.1
Albumin [g/L]	38 (36–40)	35 (31–39)	24 (20–27)	**<0.001**
Alkaline phosphatase (ALP) [U/L]	81 (70–104)	74 (61–92)	108 (85–182)	**<0.001**
Alanine aminotransferase (ALT) [U/L]	25 (18–31)	31 (20–52)	30 (19–53)	0.2
Sodium [mmol/L]	138 (136–139)	136 (134–140)	139 (136–144)	**0.012**
Potassium [mmol/L]	4.80 (4.30–5.05)	4.20 (3.80–4.55)	4.40 (4.00–4.80)	**0.005**
Chloride [mmol/L]	100.5 (97.0–102.0)	101.0 (98.0–102.0)	104.0 (100.0–108.5)	**0.004**
Bicarbonate [mmol/L]	26.0 (25.0–28.0)	24.0 (21.5–26.0)	25.0 (22.0–29.0)	0.078
Calcium [mmol/L]	2.30 (2.24–2.33)	2.26 (2.15–2.34)	2.11 (2.05–2.24)	**<0.001**
Adjusted calcium [mmol/L]	2.30 (2.26–2.37)	2.35 (2.29–2.44)	2.43 (2.35–2.56)	**0.001**
Aspartate aminotransferase (AST) [U/L]	24 (19–35)	24 (20–40)	34 (24–61)	**0.016**
Lactate dehydrogenase (LDH) [U/L]	200 (192–209)	270 (217–352)	451 (338–557)	**<0.001**
Ferritin [μg/L]	322 (197–448)	344 (192–675)	875 (623–1,776)	**<0.001**
Interleukin-6 (IL-6) [pg/mL]	3 (3–3)	25 (10–48)	76 (22–160)	**0.006**

*Bold indicates statistically significant differences with p < 0.05*.

### Global Metabolic Signature of COVID-19 Severity in Non-diabetic, Diabetic, Normotensive, and Hypertensive Groups

**Figure 2:**
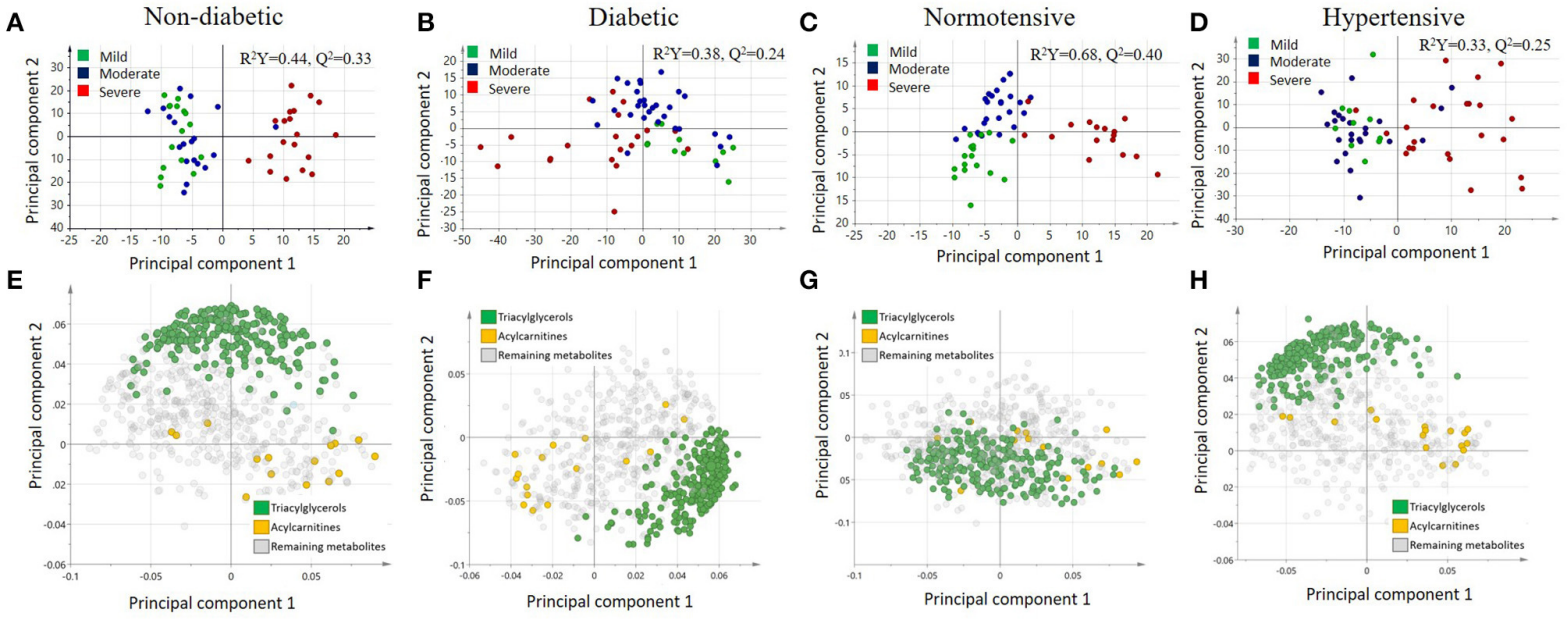
Score plots illustrating the orthogonal partial least squares discriminant analysis (OPLS-DA) for **(A)** non-diabetic, **(B)** diabetic, **(C)** normotensive, and **(D)** hypertensive COVID-19 patients as well as the corresponding loading plots for **(E)** non-diabetic, **(F)** diabetic, **(G)** normotensive, and **(H)** hypertensive COVID-19 patients.

A multivariate analysis using orthogonal partial least squares discriminant analysis (OPLS-DA) was performed to capture the combined metabolic profiles in non-diabetic, diabetic, normotensive, and hypertensive patients with varying levels of COVID-19 severity. There is a clear separation between severe and mild/moderate groups in non-diabetic patients (R2Y = 0.44, Q2 = 0.33) (Figure 2A), but there was no such separation for diabetic patients (R2Y = 0.38, Q2 = 0.24) (Figure 2B). Similarly, the score plot for normotensive (Figure 2C) patients showed a clear separation between severe and mild/moderate groups on the x-axis and between mild and moderate cases on the y-axis (R2Y = 0.68, Q2 = 0.40). However, for hypertensive patients (Figure 2D), there was no visible separation between mild and moderate groups but only between the mild/moderate and severe groups (R2Y = 0.33, Q2 = 0.25). The corresponding loading plots (Figures 2E–H) revealed an accumulation of triacylglycerols in the mild/moderate groups, whereas various acylcarnitines seemed to accumulate in the severe group across all patients.

### Metabolites Associated With COVID-19 Disease Severity in T2DM Patients

**Figure 3:**
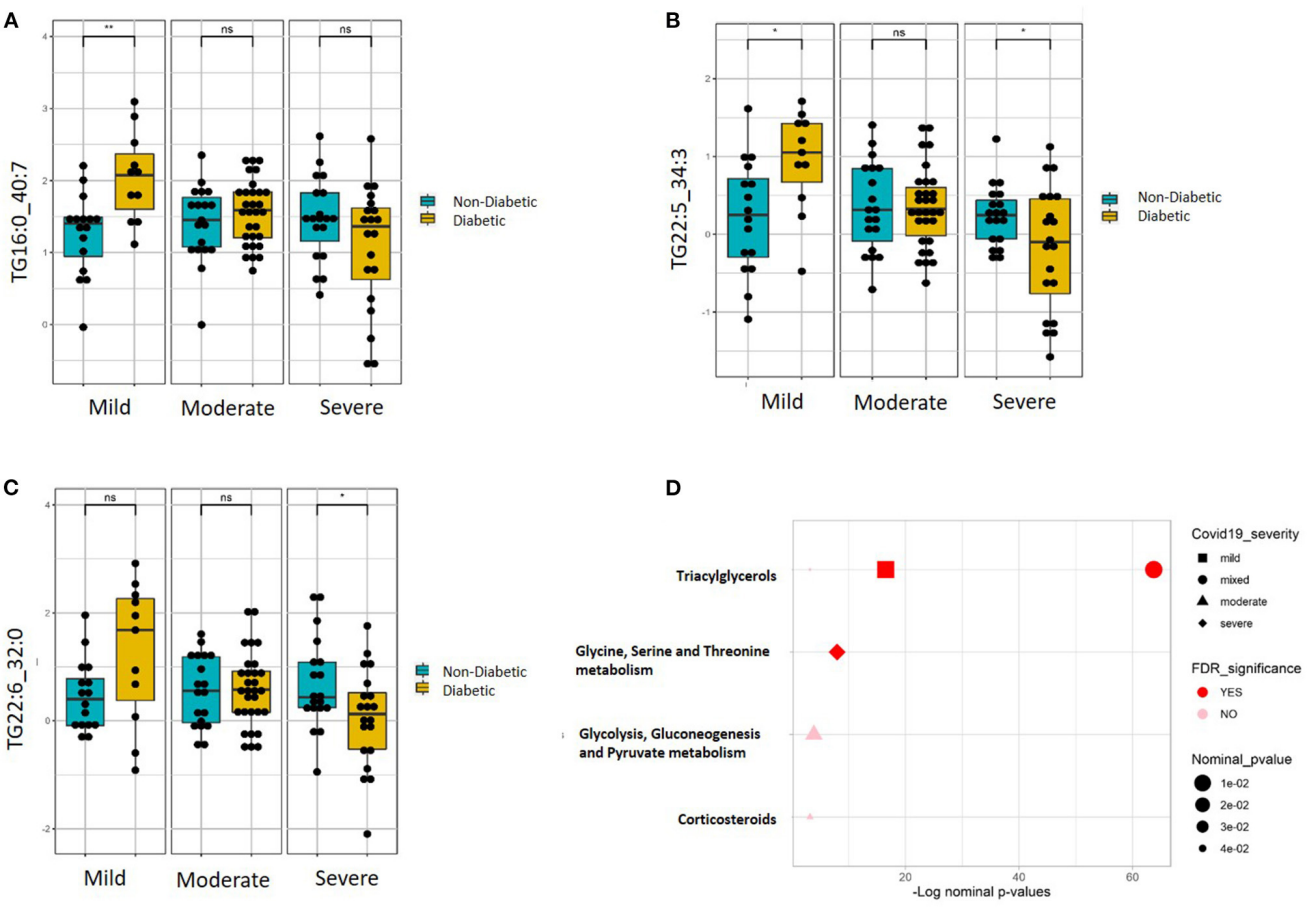
Metabolites associated with COVID-19 severity levels in diabetic and non-diabetic patients. Examples of top metabolites containing **(A)** C16:0, **(B)** C22:5, and **(C)** C22:6, which show significantly different slopes between diabetic and non-diabetic patients from regressing the given metabolite on the ordinal COVID-19 severity levels (FDR <= 0.05). **(D)** Enriched metabolite classes in terms of COVID-19 disease severity. *p ≤ 0.05, **p ≤ 0.01, ns, not significant.

Targeted metabolomics of serum samples from the 115 participants was applied to identify metabolites that best differentiated between non-diabetic and diabetic patients with increasing COVID-19 severity. Linear regression analysis revealed several significant (FDR ≤ 0.1) metabolic changes between non-diabetic and diabetic COVID-19 patients in association with severity (ordinal) (Table 2). The altered metabolic changes involved reduced levels of various triacylglycerols in the diabetic patients that varied with different levels of disease severity, including those containing the saturated fatty acid C16:0 (palmitic acid) as well as those containing the polyunsaturated fatty acids C22:5 (docosapentaenoic acid, DPA) and C22.6 (docosahexaenoic acid). Figures 3A–C shows examples of significantly reduced levels of these triacylglycerols containing palmitic acid, in terms of disease severity in diabetic patients. Enrichment analysis revealed severity-wise changes in the triacylglycerol pathways in diabetic patients compared to non-diabetic patients (FDR = 7.09 × 10^−27^). Further analysis revealed metabolites that were significantly associated with mild (Supplementary Table 1), moderate (Supplementary Table 2) and severe (Supplementary Table 3) COVID-19 in diabetic patients at nominal, but not FDR, level of significance (*p* < 0.05). Among these, triacylglycerols containing C22:5 (DPA), C22:6 (DHA), and oleic acid (C18:1) were more prevalent in mild COVID-19 diabetic patients compared to moderate and severe counterparts (Supplementary Tables 1–3). Further enrichment analyses revealed triacylglycerols as an enriched pathway in mild cases (FDR = 2.27 × 10^−06^), while severe cases were found to have enriched glycine, serine, and threonine metabolism (FDR = 0.01). Figure 3D summarizes the results of functional enrichment analyses in the mixed model (categorical), mild, moderate and severe cases.

**Table 2:** Metabolites associated with COVID-19 disease severity in diabetic patients.

**Metabolite ID**	**Pathway**	**Fold change**	**Standard error**	**Nominal *p*-value**	**FDR**
TG:22:5_34:3	Triacylglycerols	−0.69	0.18	0.0003	0.096
TG:16:0_40:7	Triacylglycerols	−0.73	0.20	0.0005	0.096
TG:16:0_38:6	Triacylglycerols	−0.63	0.18	0.0006	0.096
TG:22:6_34:3	Triacylglycerols	−0.87	0.25	0.0008	0.096
TG:16:0_38:7	Triacylglycerols	−0.65	0.19	0.0010	0.096
TG:22:6_34:2	Triacylglycerols	−0.85	0.25	0.0010	0.096
TG:18:1_38:6	Triacylglycerols	−0.57	0.17	0.0011	0.096
TG:22:5_32:1	Triacylglycerols	−0.60	0.18	0.0012	0.096
TG:22:6_34:1	Triacylglycerols	−0.82	0.25	0.0015	0.096
TG:16:0_40:8	Triacylglycerols	−0.72	0.22	0.0016	0.096
TG:18:0_34:3	Triacylglycerols	−0.74	0.23	0.0017	0.096
TG:18:0_38:6	Triacylglycerols	−0.61	0.19	0.0021	0.096
TG:22:6_32:0	Triacylglycerols	−0.89	0.28	0.0022	0.096
TG:22:5_34:2	Triacylglycerols	−0.59	0.19	0.0023	0.096
TG:20:5_34:2	Triacylglycerols	−0.78	0.25	0.0025	0.096
TG:22:6_32:1	Triacylglycerols	−0.83	0.27	0.0031	0.101

### Metabolites Associated With COVID-19 Disease Severity in Patients With Hypertension

**Figure 4:**
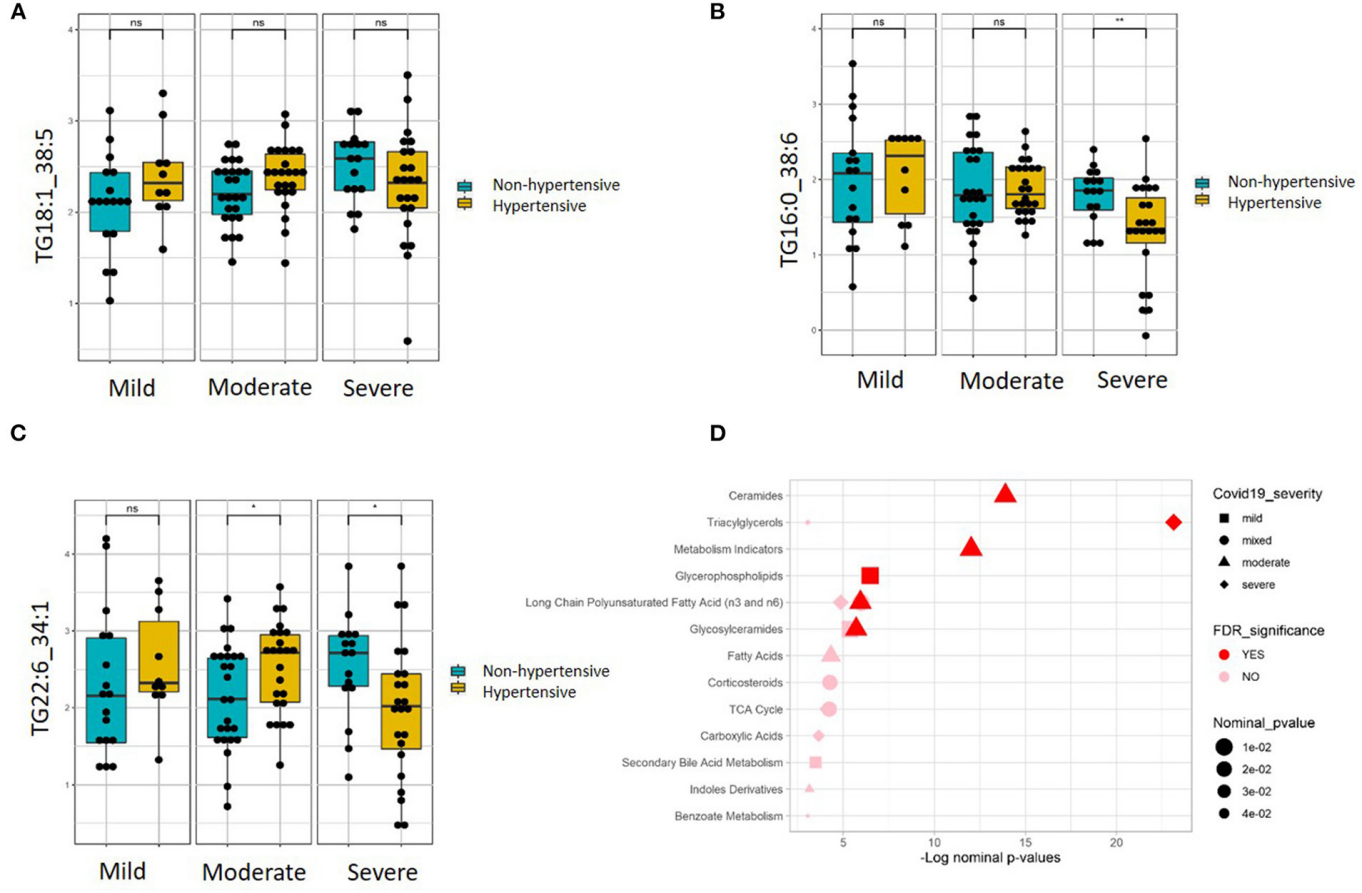
Metabolites associated with COVID-19 severity levels in normotensive and hypertensive patients. Examples of metabolites containing **(A)** C16:0, **(B)** C18:1, and **(C)** C22:6, which show significantly different slopes between hypertensive and non-hypertensive patients from regressing the given metabolite on the ordinal COVID-19 severity levels (FDR <= 0.05). **(D)** Enriched metabolite classes in terms of COVID-19 disease severity. *p ≤ 0.05, **p ≤ 0.01, ns, not significant.

Linear regression revealed a number of nominally (≤0.001) significant changes between normotensive and hypertensive COVID-19 patients in different COVID-19 severity groups (Supplementary Table 4). These changes include alterations in the levels of various triacylglycerols in the hypertensive patients with higher disease severity, including elevation in those triacylglycerols containing the saturated fatty acid C16:0 (palmitic acid) but reduction in those containing monounsaturated fatty acid C18:1 (oleic acid) and polyunsaturated fatty acid C22:6 (DHA). Figures 4A–C shows examples of the top nominally significant reduced levels of these triacylglycerols based on disease severity in hypertensive patients. Enrichment analysis revealed changes in the long-chain polyunsaturated fatty acid (n3 and n6) pathway based on disease severity in hypertensive patients compared to normotensive counterparts (FDR = 0.08). Other nominally significant pathways include corticosteroids (*p* = 0.01), TCA cycle (*p* = 0.01), triacylglycerols (*p* = 0.05), and benzoate metabolism (*p* = 0.05). Further analysis revealed metabolites associated with mild (Supplementary Table 5), moderate (Supplementary Table 6), and severe (Supplementary Table 7) cases of COVID-19 in patients with hypertension. None of these changes were significant with regard to the FDR value. Further enrichment analyses in these groups revealed that the glycerophospho-lipid (FDR = 0.05) and glucosylceramide (FDR = 0.07) pathways were enriched in mild cases, while the ceramide (FDR = 2.66 × 10^−05^), long-chain polyunsaturated fatty acid (n3 and n6) (FDR = 8.70 × 10^−05^), glucosylceramide (FDR = 0.02), and fatty acids (FDR = 0.08) pathways were enriched in moderate cases. In severe cases, only the triacylglycerol pathway was enriched pathway (FDR = 2.68 × 10^−09^). Other pathways were enriched at a nominal level of significance. Figure 4D summarizes results from functional enrichment analyses in the mixed model, mild, moderate and severe cases.

## Discussion

Severe acute respiratory syndrome coronavirus 2 (SARS-CoV-2) triggers changes in host metabolism to support its survival and replication. These alterations can influence the immune response of the host, resulting in various outcomes that range from asymptomatic infection to acute respiratory distress syndrome, vascular dysfunction, multiple organ failure, and death (16). The wide range of responses varies significantly according to many factors, including pre-existing metabolic syndrome such as T2DM and hypertension (17, 18). A number of studies have addressed the metabolic profiling of COVID-19 patients in relation to disease severity (12), ICU status (11), and various associated complications (10, 13, 14, 19). However, no studies have addressed the metabolic profiling of diabetic and hypertensive patients. Therefore, the objective of this study was to investigate the metabolic signature associated with diabetic and hypertensive COVID-19 patients in terms of varying disease severity levels.

This study aimed to identify potential diagnostic biomarkers that could also help explain the underlying molecular mechanisms responsible for increased risk in these COVID-19 patients. The identification of these mechanisms would provide early diagnostic and potentially therapeutic targets. In this study, targeted metabolomics of serum samples from COVID-19 patients with different levels of disease severity has revealed alterations in triacylglycerols containing certain fatty acids. Functional enrichment analysis revealed that changes in triacylglycerols and long-chain polyunsaturated fatty acids (n3 and n6) are associated with increased severity of COVID-19 in patients with T2DM and hypertension, with potential implications on elevated inflammatory response in these patients.

Our emerging data suggest that changes in triacylglycerols, particularly the reduction in triacylglycerols containing the long-chain saturated fatty acid palmitic acid (C16:0), were associated with increased disease severity in COVID-19 patients with T2DM. Specific saturated fatty acids, such as palmitic acid, were previously reported to significantly increase in COVID-19 patients (20). Palmitic acid is also associated with an increased risk of T2DM (21). Elevated levels of plasma non-esterified fatty acids link obesity with insulin resistance and T2DM. Recent studies have reported that long-chain saturated fatty acids such as palmitic acid can induce cellular dysfunction and lipotoxicity in various tissues, including upon insulin-producing cells, while polyunsaturated fatty acids exhibit low lipotoxic effects (22–24).

Long-chain saturated fatty acids were also shown to trigger inflammatory markers in various tissues (25), whereas n-3 fatty acids induce an anti-inflammatory effect in the immune cells (26–30). In our study, the reduced levels of palmitic acid-containing triacylglycerols in diabetic patients with severe COVID-19 suggest increased levels of its free form, the latter of which triggers an inflammatory response related to its lipotoxic effect (25–30). Our data also showed reduced levels of triacylglycerols containing C22:5 (DPA) and C22:6 (DHA) acid. Docosapentaenoic acid is converted to DHA via elongation of DPA to C24:5, followed by shortening to DHA. The protective role of DHA against metabolic syndrome is rather controversial. The anti-diabetic effect of DHA was suggested to lower the risk of cardiovascular and circulatory diseases by lowering inflammation as well as triacylglycerols levels (31, 32). However, a meta-analysis showed that neither fish consumption nor EPA/DHA consumption decreased the risk of developing diabetes except for Asian cohorts (33). The lower levels of docosapentaenoic acid and DHA-containing triacylglycerols in diabetic patients with severe COVID-19 may suggest reduced availability of its free form, hence the elevated inflammatory response.

Similar results were obtained for hypertensive COVID-19 patients, as triacylglycerols containing palmitic acid (C16:0) and DHA were reduced with increased disease severity. Palmitic acid was reported to increase the blood cholesterol levels in patients with COVID-19, perhaps due to the unsaturated fatty acid biosynthesis that could trigger upregulation of palmitic acid in infected patients (20). The Atherosclerosis Risk in Communities (ARIC) study, which monitored 16,000 adults over a period of 30 years, showed that hypertensive men had a higher proportion of cholesterol esters with elevated palmitic acid (34). The underlying mechanism is not fully understood but may reflect an association with insulin resistance and hypertension (35).

In contrast, our results showed increased levels of oleic (C18:1) acids in hypertensive patients with increased disease severity. Oleic acid was previously reported to prevent the AMPK activity reduction caused by palmitic acid, which lowers endoplasmic reticulum stress and inflammation and activates the target of rapamycin complex (mTORC)1–S6K1 pathway (15). The increased levels of oleic acid that were seen in our severe COVID-19 patients might be explained by the beneficial anti-inflammatory effects of oleic acid as well as its countering of the effects of palmitic acid.

Although in both diabetic and hypertensive patients, triacylglycerols levels seem to decrease with disease severity, it is not possible to verify whether these reduced triacylglycerole levels are byproducts or a cause of COVID-19 severity since the study lacks non-COVID-19 controls. However, both diabetes and hypertension are known to increase triacylglycerole levels (36–39) and patients with severe COVID-19 progression were reported to have higher triacylglycerole levels before the infection (40–42). Furthermore, evidence suggests that infectious diseases are associated with significant changes in lipid metabolism (41). Whether these changes are mediators or byproducts of COVID-19 severity, it remains to be investigated. Our study is limited by a small size of the cohort and male bias related to the recruitment site. Future studies focusing on validating the functional relevance of the identified metabolic biomarkers of disease progression in COVID-19 diabetic and hypertensive patients in relation to various phenotypes including antibody titers are warranted.

## Conclusions

To conclude, our emerging data highlights changes in lipid metabolism that are associated with COVID-19 severity in patients with T2DM and hypertension. The data suggests that specific long-chain unsaturated, monounsaturated, and polyunsaturated fatty acids may act as potential diagnostic and therapeutic targets. Validation of these findings in different cohorts as well as functional validation through mapping the lipids-virus interaction are warranted to convert patient-specific metabolic data into therapeutic targets.

## Data Availability Statement

The original contributions presented in the study are included in the article/Supplementary Material, further inquiries can be directed to the corresponding author/s.

## Ethics Statement

The studies involving human participants were reviewed and approved by Hamad Medical Corporation and Qatar University. The patients/participants provided their written informed consent to participate in this study.

## Author Contributions

ME is responsible for the integrity of the work as a whole. All authors contributed to sample collection, analysis, paper writing and paper review, and acceptance of final version.

## Funding

This research was funded by the Qatar National Research Fund, Grant Number NPRP11S-1212-170092.

## Conflict of Interest

The authors declare that the research was conducted in the absence of any commercial or financial relationships that could be construed as a potential conflict of interest.

## Publisher's Note

All claims expressed in this article are solely those of the authors and do not necessarily represent those of their affiliated organizations, or those of the publisher, the editors and the reviewers. Any product that may be evaluated in this article, or claim that may be made by its manufacturer, is not guaranteed or endorsed by the publisher.
